# Testing the occurrence of convergence in the craniomandibular shape evolution of living carnivorans*

**DOI:** 10.1111/evo.14229

**Published:** 2021-05-07

**Authors:** Davide Tamagnini, Carlo Meloro, Pasquale Raia, Luigi Maiorano

**Affiliations:** ^1^ Department of Biology and Biotechnologies “Charles Darwin,” University of Rome “La Sapienza,” Rome 00185 Italy; ^2^ Museum of Zoology, Sapienza Museum Centre University of Rome “La Sapienza,” Rome 00185 Italy; ^3^ Research Centre in Evolutionary Anthropology and Palaeoecology, School of Natural Sciences and Psychology Liverpool John Moores University Liverpool L3 3AF United Kingdom; ^4^ Dipartimento di Scienze della Terra, dell'Ambiente e delle Risorse University of Naples Federico II Napoli 80126 Italy

**Keywords:** Convergence, diet, ecomorphology, evolutionary trend, geometric morphometrics, skull

## Abstract

Convergence consists in the independent evolution of similar traits in distantly related species. The mammalian craniomandibular complex constitutes an ideal biological structure to investigate ecomorphological dynamics and the carnivorans, due to their phenotypic variability and ecological flexibility, offer an interesting case study to explore the occurrence of convergent evolution. Here, we applied multiple pattern‐based metrics to test the occurrence of convergence in the craniomandibular shape of extant carnivorans. To this aim, we tested for convergence in many dietary groups and analyzed several cases of carnivoran convergence concerning either ecologically equivalent species or ecologically similar species of different body sizes described in the literature. Our results validate the occurrence of convergence in ecologically equivalent species in a few cases (as well as in the case of giant and red pandas), but almost never support the occurrence of convergent evolution in dietary categories of living carnivorans. Therefore, convergent evolution in this clade appears to be a rare phenomenon. This is probably the consequence of a complex interplay of one‐to‐many, many‐to‐one, and many‐to‐many relationships taking place between ecology, biomechanics, and morphology.

The occurrence of similar traits in distantly related species is commonly known as convergence and implies that those traits are independently pushed to evolve toward a common selective optimum (Wake et al. 2011; Speed and Arbuckle 2017). Convergent evolution can be seen as an example of evolutionary trend (i.e., persistent and directional changes in the state of one or more quantitative traits, resulting in substantial changes through time—McNamara 2006), and specifically one in which multiple groups evolve to reduce their distance in the multivariate trait space (Huang et al. 2015; Stayton 2015a; Bolnick et al. 2018). Convergent evolution may also increase the similarity between distantly related species without completely obliterating the preexisting differences, leading to what is defined as “incomplete convergence” (Herrel et al. 2004; Stayton 2006; Losos 2011).

When morphology is investigated, Pigot et al. (2020) suggested that the constraints imposed by the putatively limited number of ecological niches within a clade might contribute to produce recurrent patterns of evolution toward similar morphotypes, thus resulting in iterative evolution of morphological similarities (Simpson 1944, 1953; Coxall et al. 2007; Van Valkenburgh 2007; Slater 2015). This has been observed, for instance, both in flightless birds (Wright et al. 2016; Hume and Martill 2019; Pigot et al. 2020) and in *Anolis* lizards of the Caribbean islands (Losos 1992; Mahler et al. 2013). Although there are many more examples of morphological convergence in multiple animal and plant groups, Stayton (2008) recently demonstrated using simulations that the detection of this phenomenon depends on the number of species investigated within clades in relation to the number of traits. A further consideration is the growing evidence that convergence is rare in many real case studies. For example, Grossnickle et al. (2020) only found support for incomplete convergence in the skeleton of gliding mammals (presumably constrained by strict biomechanical and physical requirements acting on nonpowered flight). Zelditch et al. (2017) similarly argued that ecomorphological convergence in the jaw shape of squirrels is rare and occurs among ecological categories that are extremely size constrained such as nut‐eating and bark‐gouging species. The authors suggested that the combination of one‐to‐many, many‐to‐one, and many‐to‐many relationships between ecology and function (which produce a complex structure of the underpinning adaptive landscape) is responsible for the rarity of convergent evolution.

The craniomandibular complex constitutes a suitable biological structure to investigate ecomorphological dynamics in mammals mainly because of the different roles played by its two components: the cranium and the mandible (Moss and Young 1960). The cranium is a functionally complex structure whose morphology is influenced by disparate demands such as protecting the brain, feeding, and agonistic behavior, as well as sensory perception (Cheverud 1981; Hallgrímsson et al. 2007). Besides, the origin of cranial bones is partly heterochronic and developmentally heterogeneous, with some originating endochondrally and others from intramembranous ossification (Sperber 2001). The mandible, in contrast, performs fewer functions mainly related to feeding (i.e., food capturing and processing—Hylander and Johnson 1994), as well as agonistic behaviors, and it is made of a single bone that develops from the simple ossification of an osteogenic membrane (Sperber 2001). Thus, one might expect that these two functionally and anatomically integrated, yet distinct, structures respond differently to evolutionary pressures, with the cranium being potentially subject to a higher number of structural and functional constraints than the mandible.

The study of the craniomandibular complex is particularly interesting in species belonging to clades with substantial phenotypic variability and disparate ecologies, such as the members of the mammalian order Carnivora (henceforth, simply called carnivorans—Ewer 1973; Gittleman 1986). Indeed, this clade represents a common model for ecomorphological investigations, which include the study of morphological convergence in relation to ecological shifts (Radinsky 1981a,b, 1982; Van Valkenburgh 1989, 1991, 2007; Figueirido et al. 2010, 2013; Meloro et al. 2015; Dumont et al. 2016; Michaud et al. 2018).

Convergence has been repeatedly detected in the morphology of both extant and fossil carnivorans (e.g., Van Valkenburgh 2007; Figueirido et al. 2010, 2013; Tseng and Wang 2011; Meloro et al. 2015). Morphological similarities in species with overlapping diets, such as the giant (*Ailuropoda melanoleuca*) and the red panda (*Ailurus fulgens*), offer some popular textbook examples of ecological convergence in phenotypic adaptations. Although radically different in body size, these two phylogenetically distant species evolved similarities in craniomandibular and appendicular morphology (e.g., wide zygomatic arches, powerful cheek teeth, expanded radial sesamoids), as well as similar physiological adaptations and developmental pathways (e.g., modifications in the amino acid metabolism and mutations in limb development genes—Hu et al. 2017), to specialize on a diet almost exclusively made of bamboo (Salesa et al. 2006; Figueirido et al. 2010). If, for pandas, convergence is found in a pair of species living in the same habitat and the same region, carnivorans provide popular examples of convergent morphological adaptations also in species that evolved in different continents. These pairs of species are commonly seen as “ecologically equivalent,” because they live in different geographical regions but occupy similar ecological niches (Lincoln et al. 1998; Biggins et al. 2011). For instance, the Eurasian raccoon dog (*Nyctereutes procyonoides*) is considered the ecological equivalent of the North American raccoon (*Procyon lotor*—Ward and Wurster‐Hill 1990), and the Malayan civet (*Viverra tangalunga*) the ecological equivalent of the Holarctic red fox (*Vulpes vulpes*—Larivière and Pasitschniak‐Arts 1996; Veron et al. 2014).

The ecological factor most frequently assumed to have produced morphological convergence in carnivorans is diet. This has led many researchers to suggest the existence of broad diet‐related ecomorphotypes such as pack hunters (i.e., the spotted hyena and large wild canids—Meloro et al. 2015) or durophagous feeders (common among ursids, mustelids, and hyaenids—Figueirido et al. 2013). However, Meloro et al. (2015) also observed that morphological convergence in the mandible of carnivorans heavier than 7 kg is rare when comparing species belonging to the same dietary category, probably because of a rapid diversification in terms of size and a less evolutionary malleable shape occurring in this clade. If confirmed, this would suggest that overlapping diets may contribute to morphological convergence but do not necessarily lead to it.

Craniomandibular convergence in carnivorans has already been tested by several authors comparing linear measurements or qualitative morphological features (Gaubert et al. 2005) as well as by applying geometric morphometrics (GMM—Figueirido et al. 2010, 2013; Meloro et al. 2015, 2017). However, despite these many studies on the Carnivora, convergence has never been extensively explored in this clade using a large taxonomic sample representative of its vast ecomorphological disparity. In this study, we assessed the presence and strength of convergence in the shape of the craniomandibular complex of living carnivorans, using GMM and three different pattern‐based (i.e., able to detect patterns regardless of the processes behind them) indices designed for detecting the occurrence of retained and/or evolved similarity: C1 (Stayton 2015b), θ (Castiglione et al. 2019), and Wheatsheaf (Arbuckle et al. 2014) metrics. More precisely, we investigated morphological convergence by grouping species based on the type of prevalent food in their diet. Then, we considered several cases of potential morphological convergence by focusing on ecologically equivalent species of broadly similar body size or sympatric taxa with strong similarity in diet and habitat but large differences in size.

## Materials and Methods

### DATA COLLECTION, SAMPLES, GMM, AND PHYLOGENY

Cranial and mandibular photographic samples were collected by the same operator (CM) using a digital SLR Nikon D40 equipped with a Nikkor 70–200 mm lens, at a focal length of 100 mm. A horizontal tripod was employed to position the camera above the specimens to hold the camera still and minimize photographic distortions (e.g., Muir et al. 2012). A spirit level was used to verify that the camera and the specimen were approximately parallel.

Samples consist of 529 crania photographed in ventral view and 554 mandibles in lateral view. They represent more than 60% of the existing carnivoran species diversity (188 out of 296). The taxonomy adopted in this study followed the IUCN Red List website (https://www.iucnredlist.org). Almost all specimens, except for a few (i.e., three crania and three mandibles of *Mirounga leonina*) made available by the Falkland Islands Elephant Seal Research Group (http://eleseal.org/), came from museum collections including National Museums of Scotland (Edinburgh), World Museum (Liverpool), Natural History Museum (London), Kenya National Museums (Nairobi), and Royal Museum for Central Africa (Tervuren). Sample compositions are detailed in Table S1 and a full list with catalogue numbers is available upon request.

All individuals were adults, as assessed by the presence of complete dentition and the fusion of cranial sutures. For each species, a minimum of one cranium and one mandible was collected, including both sexes whenever available. When multiple specimens belonging to the same species were available, morphological data were averaged within species, obtaining pooled‐sex data. Using a few individuals to represent a species is never ideal (Cardini 2020), but nonetheless feasible in macroevolutionary analyses with wide phylogenetic scope (e.g., Drake and Klingenberg 2010; Meloro and O'Higgins 2011).

**Figure 1:**
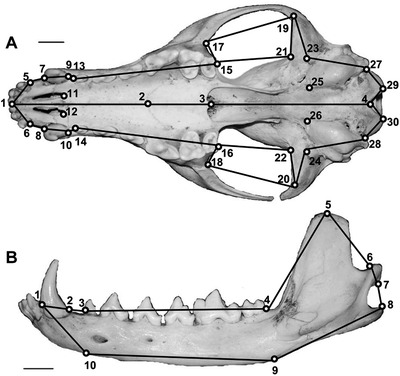
Landmark configuration, together with the wireframe, on cranium (A) and mandible (B) of red fox (*Vulpes vulpes*). Scale bar is 1 cm.

The two‐dimensional landmark digitization was performed using the software TPSDig (version 2.21—Rohlf 2015) by a single operator (DT) to avoid interoperator biases (e.g., misinterpretation of landmark definitions). Landmark configurations for the cranium and the mandible are shown in Figure 1, and their anatomical definitions are provided in Table 1. The selected configuration of landmarks generally followed Meloro and O'Higgins (2011) and Tamagnini et al. (2017) to describe the main morphofunctional regions of the craniomandibular complex. This configuration ensured the anatomical correspondence of homologous landmarks among all the specimens without particular references to the postcanine dentition, except for the length of the tooth row, because premolars and molars are indistinguishable in the seals and the walrus.

**Table 1:** Definitions of the anatomical landmarks

**Cranium**
Midplane
1 Most anterior point on premaxilla
2 Meeting point of maxilla and palatine
3 Posterior endpoint of palatine
4 Most anterior point on the rim of the foramen magnum
Bilateral
5–6 Posteromedial point on the alveolar margin of the last upper incisor
7–8 Anteromedial point on the alveolar margin of the canine
9−10 Posteromedial point on the alveolar margin of the canine
11–12 Most posterior edge of the palatine foramen
13–14 Anteromedial point on the alveolar margin of the premolar starting the upper premolar row
15−16 Posteromedial point on the alveolar margin of the last tooth of the upper jaw
17–18 Anterior point of maximum curvature on the interior side of the zygomatic arch
19−20 Posterior point of maximum curvature on the interior side of the zygomatic arch
21–22 Interior side margin of the glenoid fossa
23–24 Medial side margin of the glenoid fossa
25–26 Meeting point of basioccipital, basisphenoid, and tympanic bulla
27–28 and 29–30 Edges of the occipital condyle

**Mandible**
1 Most anterior point on the alveolar margin of the canine
2 Most posterior point on the alveolar margin of the canine
3 Anterior point on the alveolar margin of the first tooth in the premolar row
4 Posterior point on the alveolar margin of the last tooth in the molar row
5 Tip of the coronoid process
6–7 Maximum depth of the condylar process
8 Most lateral extreme point of the angular process
9 Vertical projection of 4 perpendicular to the line defined by (3–4)
10 Vertical projection of 3 perpendicular to the line defined by (3–4)

To remove nonshape variation from two dimensional Cartesian coordinates of landmarks, we employed the Procrustes superimposition (Rohlf and Slice 1990; Adams et al. 2004, 2013) using the software MorphoJ (version 1.06d; Klingenberg 2011). This procedure consists of three steps: (1) the standardization of size, (2) the removal of translational variation, and (3) the minimization of rotational differences (Rohlf and Slice 1990). Because we are using two‐dimensional measurements of three‐dimensional structures, the flattening of the third dimension inevitably introduces an error (Roth 1993). However, previous studies on crania and mandibles of marmots and living equids (Cardini 2014; Cardini and Chiapelli 2020) suggested that results are likely to be robust to the error of two‐ to three‐dimensional approximation, as long as landmarks are approximately coplanar (as in our data) and differences relatively large, as typical of macroevolutionary analyses.

The background for comparative analyses was provided by a molecular phylogeny from the 10KTrees project (Arnold et al. 2010). This phylogeny is a consensus based on 14 mitochondrial genes, 14 autosomal genes, and one gene from the Y‐chromosome. The node ages were inferred using 16 fossil calibration points, extracted from the Paleobiology Database (http://paleodb.org).

### MORPHOLOGICAL CONVERGENCE

**Figure 2:**
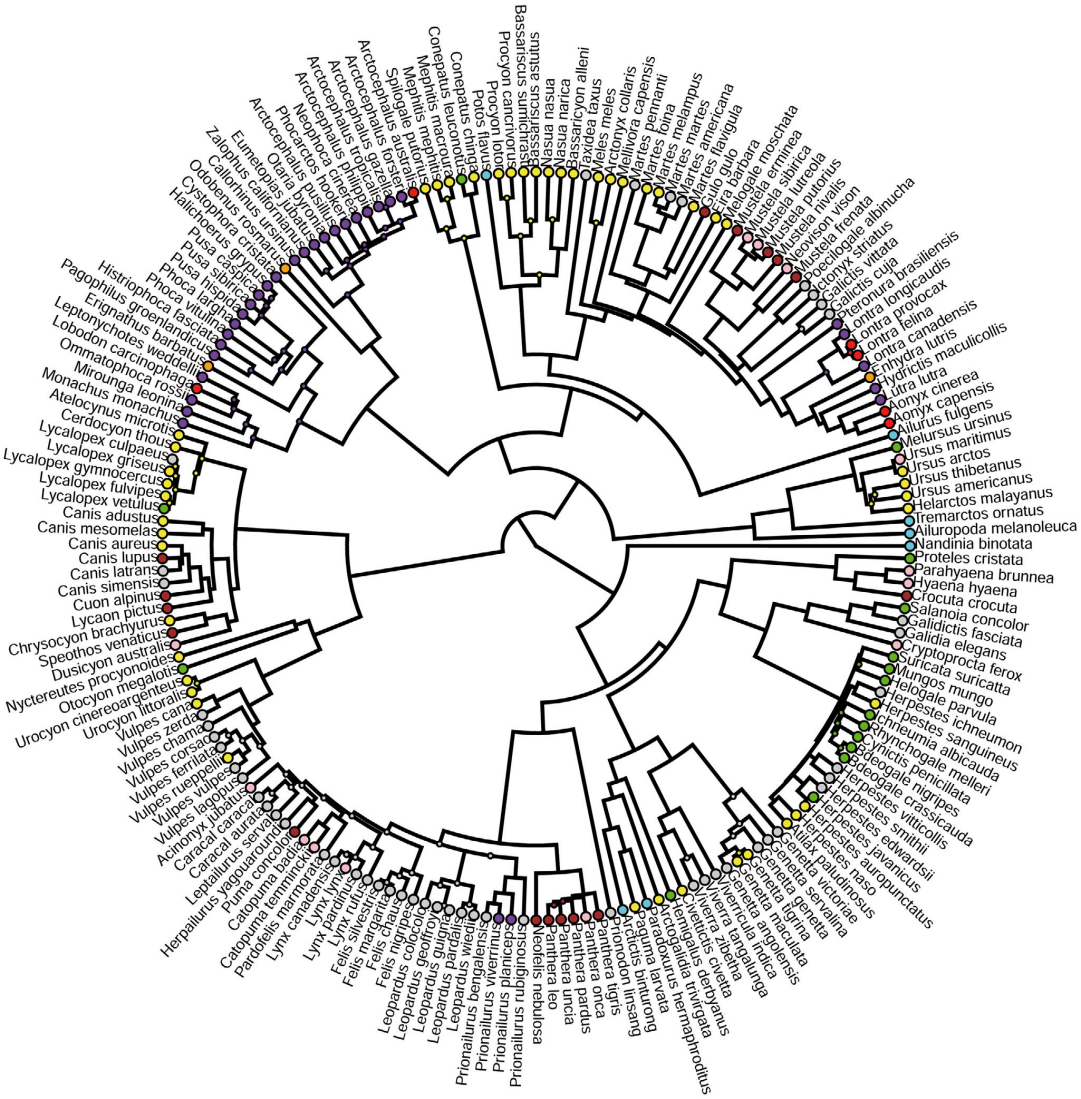
Circular dendrogram representing the 10KTrees phylogeny (Arnold et al. 2010), and showing the distribution of each food item category in living carnivorans. Large prey hunters are represented in brown, medium prey hunters in pink, small prey hunters in grey, herbivores/frugivores in turquoise, insectivores in green, piscivores in purple, crustacivores in red, molluscivores in orange, and omnivores in yellow.

To test for convergence in dietary groups, each species was ascribed to one out of nine mutually exclusive dietary categories following Christiansen and Wroe (2007) for terrestrial carnivorans, and Jones et al. (2013) for pinnipeds. The categories are as follows: large prey hunters, medium prey hunters, small prey hunters, herbivores/frugivores, insectivores, piscivores, crustacivores, molluscivores, and omnivores (Fig. 2). These are all the possible ecological groups obtained adopting a dietary categorization based on the main food item consumed by living carnivorans. Large, medium, and small prey hunters were distinguished based on the comparison between the size of the predator and the size of its most common prey. Omnivores included species relying almost in similar proportions on two or more food items. Whenever the attribution of a species was not provided in Christiansen and Wroe (2007) or Jones et al. (2013) or it was uncertain, we decided the most likely dietary group relying on the information available in the *Handbook of the Mammals of the World ‐ Volumes 1* and *4* (Wilson and Mittermeier 2009, 2014, and references therein).

As anticipated, besides testing convergence among species within each dietary group, we also explored whether convergence is supported in cases of species considered ecologically equivalent in different biogeographical regions (i.e., red fox—Malayan civet, raccoon dog—raccoon, Iberian lynx—fossa, and spotted hyena—wolverine) or ecologically similar but living in sympatry thanks to large differences in body size (i.e., giant panda—red panda)

### C1, θ, AND WHEATSHEAF INDEX

To test for the strength of shape convergence, we computed three different metrics designed for detecting the occurrence of retained and/or evolved similarity: C1, θ, and Wheatsheaf index.

C1 is a distance‐based measure “representing the proportion of the maximum distance between the putatively convergent species that has been ‘closed’ by subsequent evolution, and thus ranges from 0 to 1 as convergence increases” (Stayton 2015b, p. 2144). For instance, C1 = 0.5 indicates that the convergent species closed 50% of the maximum phenotypic distance between them. To assess significance, this approach simulates evolution via Brownian motion (i.e., null hypothesis) using the input tree and parameters derived from the observed data, returning a C1 metric for each simulation and calculating the *P*‐value from the number of times the simulated value exceeds the observed value. C1 metric is designed for detecting the occurrence of evolved similarity (i.e., convergence). C1 was computed and tested using the functions *convrat* and *convratsig*, embedded in the package *convevol* (Stayton 2015b).

θ is the average angle between the phenotypic vectors of putatively convergent species in the multivariate shape space (Castiglione et al. 2019). The cosine of angle θ represents the correlation coefficient between these vectors (Zelditch et al. 2012). Thus, θ is a measure of the resemblance between the phenotypes (Adams and Collyer 2009), which, under a Brownian Motion (BM) model, is expected to decrease proportionally to the time since divergence from a common ancestor. The test estimates whether the mean time‐distance‐standardized θ scores between all pairs of species evolving under a given state (e.g., dietary category) are lower than expected under BM, which would indicate retained and/or evolved similarity. Time‐distance‐standardized θ was implemented with the function *search.conv*, embedded in the package *RRphylo* (Castiglione et al. 2018, 2019).

The third and last metric we used is the Wheatsheaf index. This index is the ratio between the average phylogenetically corrected phenotypic distance computed for the entire sample to the same distance calculated only for the putatively convergent species (e.g., species in the same dietary category). Similar to C1, this index relies on phenotypic distances, whereas θ relies on angles between phenotypic vectors. To assess significance for the Wheatsheaf index, we followed the bootstrapping approach of Arbuckle et al. (2014, p. 687), which “resamples the tips of the tree along with their trait values and thus obtains a distribution of possible Wheatsheaf indices given the phylogeny and the trait values for each species. The P‐value is equal to the proportion of bootstrap samples that are greater than or equal to the value of the index calculated from the original data set.” As discussed in previous studies (Stayton 2015b; Arbuckle and Speed 2016), higher values of this index indicate not only a greater degree of clustering among the convergent taxa, but also greater distinctiveness of them, making it appropriate to test for “incomplete convergence,” but potentially conflating convergence with retained similarity. For this reason, although using only the Wheatsheaf index (as well as θ) is inadvisable to assess if a group underwent convergent evolution, combining this metric with others more strictly designed for detecting the occurrence of convergence (e.g., C1) might allow researchers to distinguish between cases of convergence and retained similarity (e.g., phases of reduced or null evolutionary rate). Wheatsheaf index was computed and tested using the functions *windex* and *test.windex*, embedded in the package *windex* (Arbuckle and Minter 2015).

C1, θ, and Wheatsheaf index were applied to each of the nine dietary categories adopted in this study (Fig. 2) to test for the presence of shape convergence in our sample. Then, these metrics were further applied to test convergence on the cases concerning either ecologically equivalent species or ecologically similar species of different body sizes. All analyses employed comparative tests using the 10Ktrees phylogeny as an estimate of evolutionary relationships. The significance of each test was assessed performing 1000 simulations against random expectations following Arbuckle et al. (2014), Stayton (2015b), and Castiglione et al. (2019). Following the example of Maiorano et al. (2008), we also adopted a multiple testing correction metric, the *Q*‐value (Storey 2002; Storey and Tibshirani 2003; Storey et al. 2004), to take into account the simultaneous implementation of several tests, which could inflate type I errors. The *Q*‐value metric is also suitable for cases where a dependency exists between a portion of the performed tests (Benjamini and Yekutieli 2001). All significance tests were carried out at the α = 0.05 level. Morphological data, R code, and phylogeny used in this study are provided as Supporting Information.

## Results

### TESTS IN DIETARY CATEGORIES

**Figure 3:**
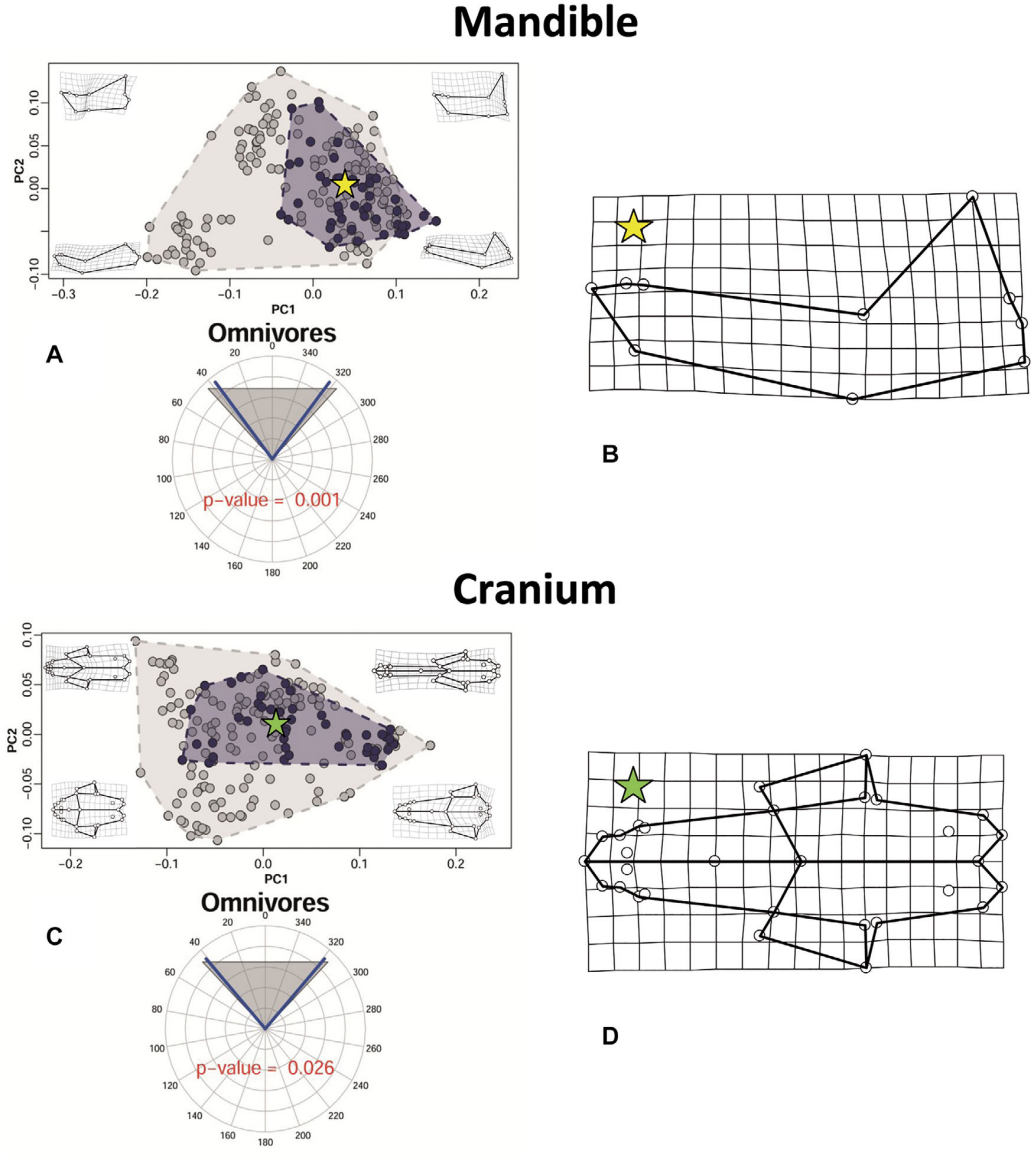
Scatterplots of mandibular (A) and cranial (C) shape variation summarized by PC1 (51.4% of variance explained for the mandible and 47.8% for the cranium) and PC2 (20.7% of variance explained for the mandible and 20.0% for the cranium). Gray convex hulls contain all the sampled species, whereas blue convex hulls contain the omnivore carnivorans. The circular plots report the mean time‐distance‐standardized θ, between the species set to converge (blue lines) and the range of random angles expected under the Brownian Motion (gray shaded area). The *P*‐value for the time‐distance‐standardized θ, test is printed within the circular plots. Deformation grids and wireframes show the shape deformation corresponding to each quadrant of shape space. Yellow and green stars represent, respectively, the position of mandibular (B) and cranial (D) consensus shapes of omnivores in the shape space.

For the mandible, C1 ranged from 0 to 0.123 (Table 2), with none of the tests reaching significance, which suggests a lack of strong evidence for convergence in any of the dietary categories we tested. Mandibular time‐distance‐standardized θ ranged, depending on the dietary group, from 0.615 to 1.035 (Table 2). Only omnivore carnivorans reached significance in both their mandibular *P*‐ and *Q*‐values regarding this metric (Fig. 3). This indicates that, within omnivores, there is more retained and/or evolved similarity than expected using a BM model of evolution. Finally, Wheatsheaf indices ranged from 0.501 to 1.736 in the mandible for all the ecological categories (Table 2). Small prey hunters and omnivores returned significant *P*‐values in the mandible regarding the Wheatsheaf index, whereas only omnivores returned a significant *Q*‐value: this outcome indicates the occurrence of retained and/or evolved similarity in omnivore carnivorans and, less convincingly, in small prey hunters.

**Table 2:** Mandibular C1, time‐distance‐standardized θ, and Wheatsheaf index scores, *P*‐values, and *Q*‐values relative to the dietary categories adopted to test the occurrence of shape convergence. Significant *P*‐values and *Q*‐values at α = 0.05 are underlined

	C1			Time‐distance‐standardized θ			Wheatsheaf index		
Dietary category	Score	*P*‐value	*Q*‐value	Score	*P*‐value	*Q*‐value	Score	*P*‐value	*Q*‐value
Herbivores/Frugivores	0.046	0.803	0.999	0.660	0.111	0.500	1.736	0.069	0.373
Insectivores	0.106	0.219	0.593	1.035	0.984	0.999	0.824	0.105	0.500
Crustacivores	0.104	0.409	0.919	0.993	0.871	0.999	0.831	0.148	0.510
Molluscivores	0.000	0.999	0.999	1.018	0.866	0.999	0.746	0.872	0.999
Piscivores	0.066	0.891	0.999	0.615	0.287	0.674	0.501	0.998	0.999
Omnivores	0.123	0.230	0.593	0.718	0.001	0.011	1.226	0.001	0.011
Large prey hunters	0.091	0.651	0.999	0.779	0.539	0.999	0.929	0.594	0.999
Medium prey hunters	0.074	0.921	0.999	0.897	0.915	0.999	1.086	0.150	0.510
Small prey hunters	0.091	0.731	0.999	0.740	0.171	0.513	0.928	0.021	0.142

Cranial C1 scores ranged from 0.006 to 0.130 (Table 3), with only large prey hunters being significant (both for *P*‐ and *Q*‐values). Cranial time‐distance‐standardized θ ranged from 0.686 to 3.560 for all the dietary categories (Table 3). Only omnivore carnivorans reached significance in their cranial *P*‐value regarding this metric (Fig. 3), whereas none of the *Q*‐values was significant. Finally, Wheatsheaf index scores ranged, for cranial shape, from 0.631 to 1.822 (Table 3). Insectivores, omnivores, and medium prey hunters reached significance in both their cranial *P*‐ and *Q*‐values regarding this metric, whereas herbivores/frugivores returned only a significant *P*‐value.

**Table 3:** Cranial C1, time‐distance‐standardized θ, and Wheatsheaf index scores, *P*‐values, and *Q*‐values relative to the dietary categories adopted to test the occurrence of shape convergence. Significant *P*‐values and *Q*‐values at α = 0.05 are underlined

	C1			Time‐distance‐standardized θ			Wheatsheaf index		
Dietary category	Score	*P*‐value	*Q*‐value	Score	*P*‐value	*Q*‐value	Score	*P*‐value	*Q*‐value
Herbivores/Frugivores	0.045	0.653	0.999	0.686	0.169	0.513	1.822	0.014	0.108
Insectivores	0.066	0.723	0.999	0.959	0.874	0.999	0.960	0.001	0.011
Crustacivores	0.035	0.881	0.999	0.973	0.831	0.999	0.721	0.285	0.674
Molluscivores	0.006	0.782	0.999	0.760	0.603	0.999	0.660	0.990	0.999
Piscivores	0.049	0.962	0.999	3.560	0.998	0.999	0.631	0.145	0.510
Omnivores	0.104	0.231	0.593	0.746	0.026	0.156	1.133	0.001	0.011
Large prey hunters	0.130	0.001	0.011	0.864	0.857	0.999	1.032	0.151	0.510
Medium prey hunters	0.062	0.848	0.999	0.839	0.775	0.999	1.265	0.003	0.027
Small prey hunters	0.084	0.692	0.999	0.811	0.691	0.999	0.842	0.465	0.999

### TESTS IN SELECTED CASES OF ECOLOGICALLY EQUIVALENT SPECIES AND SYMPATRIC SPECIES WITH SIMILAR ECOLOGY BUT LARGE SIZE DIFFERENCES

**Figure 4:**
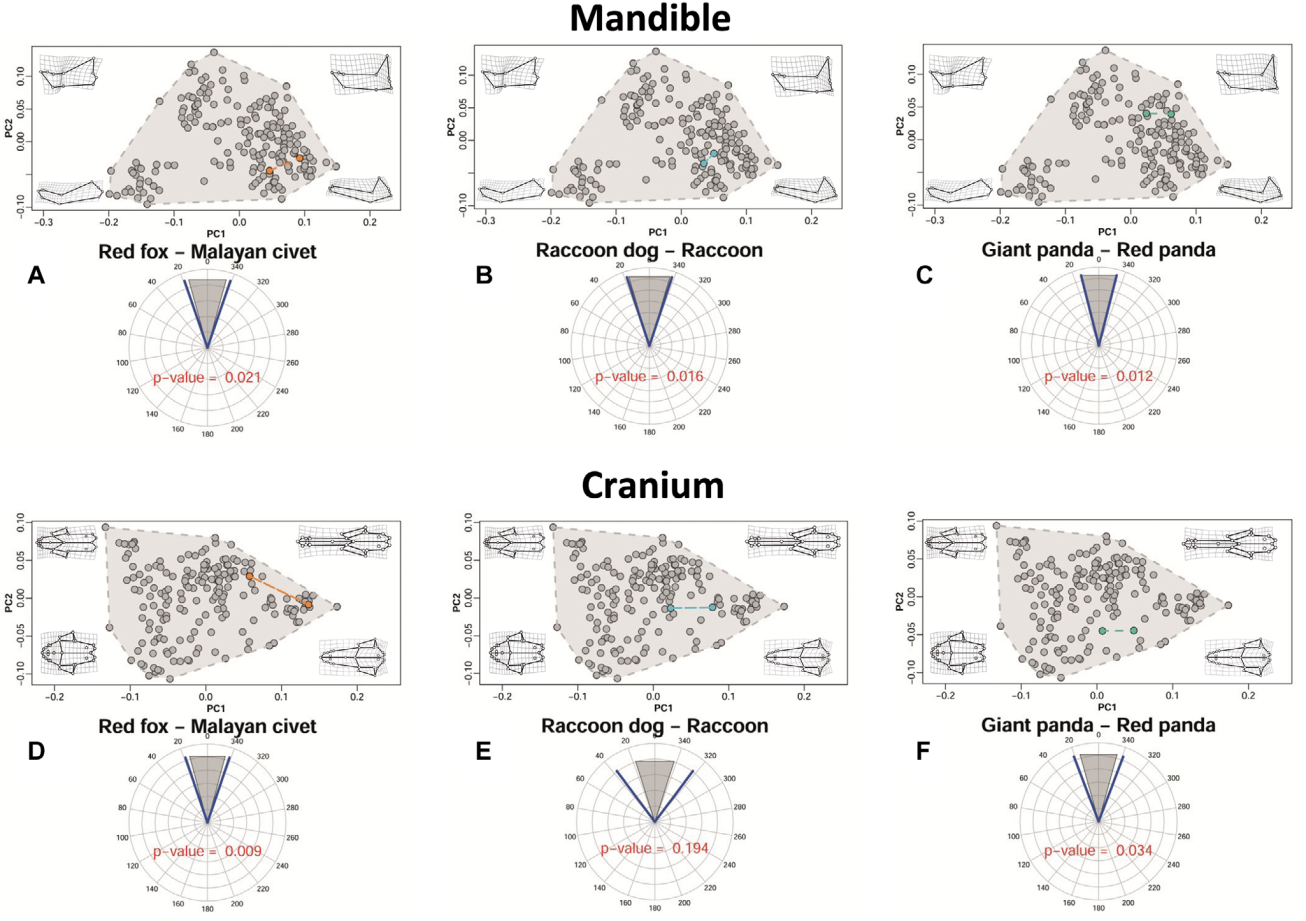
Scatterplots of mandibular (A–C) and cranial (D–F) shape variation summarized by PC1 and PC2. Gray convex hulls contain all the sampled species, whereas orange, light blue, and green convex hulls, respectively, contain the red fox—Malayan civet, the raccoon dog—raccoon, and the giant panda—red panda cases. The circular plots report the time‐distance‐standardized θ, between the species set to converge (blue lines) and the range of random angles expected under the Brownian Motion (gray shaded area). The *P*‐value for the time‐distance‐standardized θ, test is printed within the circular plots. Deformation grids and wireframes show the shape deformation corresponding to each quadrant of shape space.

In the selected cases of species with broadly similar ecological niches living either in separate biogeographical regions or sympatrically thanks to large body size differences, mandibular C1 scores were greater than 0.305 (min score = 0.305, max score = 0.598) for all the considered cases included in Table 4. The cases red fox—Malayan civet, giant panda—red panda, and raccoon dog—raccoon reached significance in both *P*‐ and *Q*‐values in the mandible regarding the C1 metric, whereas the case Iberian lynx—fossa returned only a marginally significant *P*‐value. Mandibular shape time‐distance‐standardized θ ranged from 0.220 to 0.520 (Table 4). The red fox—Malayan civet, raccoon dog—raccoon, and spotted hyena—wolverine, as well as giant panda—red panda, were all (Fig. 4) significant in terms of both *P*‐ and *Q*‐values. Finally, Wheatsheaf indices for the mandible ranged from 1.914 to 10.348 (Table 4), but significance (in both *P*‐ and *Q*‐values) was reached only in the case raccoon dog—raccoon.

**Table 4:** Mandibular (upper half) and cranial (lower half) C1, time‐distance‐standardized θ, and Wheatsheaf index scores, *P*‐values, and *Q*‐values relative to the list of cases concerning either ecologically equivalent species or ecologically similar species of different body sizes selected from the literature to test the occurrence of shape convergence. Significant *P*‐values and *Q*‐values at α = 0.05 are underlined

	C1				Time‐distance‐standardized θ			Wheatsheaf index	
Mandible	Score	*P*‐value	*Q*‐value	Score	*P*‐value	*Q*‐value	Score	*P*‐value	*Q*‐value
Red fox—Malayan civet	0.497	0.001	0.005	0.232	0.021	0.033	6.813	0.162	0.129
Raccoon dog—Raccoon	0.598	0.001	0.005	0.245	0.016	0.033	10.348	0.009	0.029
Iberian lynx—Fossa	0.305	0.050	0.061	0.520	0.294	0.173	1.914	0.251	0.154
Spotted hyena—Wolverine	0.386	0.059	0.068	0.241	0.018	0.033	5.849	0.252	0.154
Giant panda—Red panda	0.445	0.001	0.005	0.220	0.012	0.032	3.090	0.155	0.129

	C1				Time‐distance‐standardized θ			Wheatsheaf index	
Cranium	Score	*P*‐value	*Q*‐value	Score	*P*‐value	*Q*‐value	Score	*P*‐value	*Q*‐value
Red fox—Malayan civet	0.289	0.099	0.093	0.230	0.009	0.029	3.424	0.680	0.361
Raccoon dog—Raccoon	0.156	0.208	0.147	0.497	0.194	0.147	3.736	0.148	0.129
Iberian lynx—Fossa	0.169	0.238	0.154	0.614	0.351	0.200	2.528	0.073	0.078
Spotted hyena—Wolverine	0.314	0.049	0.061	0.404	0.089	0.089	5.525	0.212	0.147
Giant panda—Red panda	0.322	0.020	0.033	0.322	0.034	0.049	1.877	0.444	0.244

For the same cases used in the tests on mandibular shape, cranial C1s ranged from 0.156 to 0.322 (Table 4). With C1, the case giant panda (red panda) was significant for both *P*‐ and *Q*‐values, whereas the spotted hyena (wolverine) test was marginally significant only for the *P*‐value. Cranial time‐distance‐standardized θ ranged from 0.230 to 0.614 (Table 4), with only the red fox (Malayan civet) and giant panda (red panda) significant using both *P*‐ and *Q*‐values (Fig. 4). Cranial Wheatsheaf indices were larger than 1.877 (range = 1.877–5.525; Table 4), but none of them reached significance.

## Discussion

### IS THERE SHAPE CONVERGENCE IN THE CARNIVORAN CRANIOMANDIBULAR COMPLEX?

Our results support three main conclusions: (1) retained similarity occurs in omnivores (significant θ and Wheatsheaf index, but nonsignificant C1 and thus no clear evidence of convergence), (2) compelling evidence of convergence within dietary classes is very rare (i.e., only cranial shapes of large prey hunters converge as suggested by their significant C1, whereas mandibular shapes do not converge in any dietary class), and (3) two cases of ecologically equivalent species (i.e., red fox—Malayan civet; raccoon dog—raccoon) converge only in mandibular shape and one case of ecologically similar species of different body sizes converges in both cranial and mandibular shape (i.e., giant and red pandas).

Omnivores tend to cluster around a mean shape (Fig. 3), which has an elongated rostrum and relatively long tooth rows in both the upper and lower jaw. This is often associated with a full dental formula (Ewer 1973), a condition common in the small‐sized species belonging to the carnivoran stem group, such as those of the genus *Gustafsonia* (Tomiya and Tseng 2016, but see Werdelin 1996 for different morphologies in the carnivoran stem group). Although our results could lead to suppose the occurrence of convergent evolution in omnivore carnivorans at first, the fact that the volume of shape space occupied by omnivores is considerably large in relation to the overall shape space occupied by all species (Fig. 3), together with the lack of significance for their C1s, suggests the occurrence of evolutionary conservatism as a more likely scenario (i.e., closely related species more similar than would be expected based on their phylogenetic relationships—Losos 2008; Moen et al. 2013). According to this hypothesis, a common ancestor of omnivore carnivorans evolved the omnivore condition (that therefore arose only once in this group) and its descendants represent just a continuation of a successful morphotype. This scenario supports Simpson (1944, 1953) who suggested that evolution largely occurs within relatively narrow adaptive zones, because those wandering too far from the peaks of an adaptive zone are “weeded out by selection, whereas new zones are colonized when rapid bursts of evolution propel a species across the selectively disadvantageous space between zones” (Polly 2008, p. 3). In particular, our results are compatible with the existence of an omnivore adaptive zone in the craniomandibular shape evolution of living carnivorans, with other specialized species emerging from this region of the multivariate shape space. A similar pattern might explain how pinnipeds moved toward a progressively more specialized aquatic lifestyle and evolved a remarkably distinctive ankle shape, hugely dissimilar from those of their closer terrestrial relatives among the caniforms (Polly 2008). In contrast, terrestrial carnivorans, despite specializing for different types of terrestrial locomotion, largely retained a broadly similar foot bone morphology. This, together with other plesiomorphies, contributed to mislead taxonomists into splitting the carnivorans into pinnipeds and fissipeds, a subordinal classification no longer valid because of the paraphyletic state of the fissipeds (Arnason et al. 2007).

When it comes to the species that preponderantly consume a single food item, diet‐related convergence is supported in large prey hunters, but only for cranial shape. Many other groups (e.g., insectivores, medium prey hunters) showed significant *P*‐values and/or *Q*‐values only according to the Wheatsheaf index. Because this score is also influenced by a permanent condition of reduced (or null) evolutionary rate, our findings suggest that this outcome is the product of an extended phase of reduced shape change occurring in these categories. The discrepancy between the results of the tested metrics might be produced by the occurrence of multiple morphological optima for species belonging to the same ecological category (i.e., a phenomenon known as many‐to‐one mapping of form to function—Alfaro et al. 2005; Wainwright et al. 2005; Collar et al. 2014; Sansalone et al. 2020). It might be a consequence of different developmental constraints taking place in phylogenetically distant clades. Recent studies demonstrated that shifts from hypo‐ to hypercarnivory and vice versa are mainly due to variations in both the snout length and the dentition in canids, contrary to most other carnivoran groups in which these transitions are due to changes occurring in the dentition (Van Valkenburgh 1991; Holliday and Steppan 2004; Slater et al. 2009; Damasceno et al. 2013; Machado et al. 2018; Machado 2020). Otherwise, this pattern can be a product of ecological variation that was not accounted for in our categorization such as the impact of selective factors other than diet on craniomandibular shape evolution of these categories.

All the metrics relative to the remaining dietary categories (e.g., piscivores, molluscivores) were not significant, therefore indicating that neither convergence nor conservatism is likely to have impacted the shape variation of these groups through time. This outcome can be produced by the occurrence of neutral or divergent evolution because these patterns represent the null hypothesis of the employed metrics (Arbuckle et al. 2014; Stayton 2015b; Castiglione et al. 2019). For example, molluscivore carnivorans include species that rely on alternative strategies to feed on invertebrates protected by a hard shell. These strategies range from suction‐feeding (e.g., walrus) and, even more commonly, shell‐crushing (e.g., sea otter), to using the mandible as an anchor to dislodge hard‐shelled organisms from hard substrates, as it likely happened in the extinct marine arctoid *Kolponomos* (Tseng et al. 2016). Thus, independent evolution of similar diets can either produce convergence but can also, and probably more commonly among carnivorans, push species toward different directions in the craniomandibular multivariate trait space (Boessenecker 2012, 2017; Timm‐Davis et al. 2015; Radinsky 1981b).

If tests within broad dietary groups were mostly and consistently nonsignificant, evidence for evolutionary convergence was stronger when we compared ecologically equivalent species or cases with large size differences but very similar diets (i.e., the giant panda and red panda). This is especially evident for the mandible (with three instances of significance using both time‐distance‐standardized θ and C1) and is not incompatible with the mainly negative findings from the tests on dietary groups. As briefly mentioned, the set of tests on diet investigates the average degree of clustering within a group. Because of this averaging, even when results are negative (no support for conservatism/convergence on average) in a group, one cannot exclude that specific pairs of species within that same group could, nevertheless, be convergent. This is clearly the case of the giant and the red pandas (strongly convergent) within the herbivores/frugivores, which are for the rest hardly showing any clustering. Simply, the specific case is “lost” when results are averaged across all members of that group, a fairly obvious point but still one to bear in mind when assessing convergence: the level of the analysis is crucial and large‐scale studies might miss important details. Our analyses indicate that convergence occurred three times in ecologically equivalent species or in cases with large size differences but very similar diets, involving in the first case two small prey hunters (red fox and Malayan civet), in the second case two omnivore species (North American raccoon and raccoon dog) and in the last case two herbivore/frugivore species (giant and red pandas). Therefore, ecological equivalence might produce convergence within the same dietary category in many different carnivoran ecomorphotypes. This is coherent with previous micro‐ and macroevolutonary studies in mammals (e.g., convergence between ecologically equivalent bank voles in Southern Eurasia—Ledevin et al. 2018; convergence between Afrotheria and Laurasiatheria—Gheerbrant et al. 2016). Our results also suggest that convergence might happen, to a certain degree, even in ecologically similar species of hugely different size, as confirmed by the case of giant and red pandas.

### COMPARISON WITH PREVIOUS CONVERGENCE STUDIES ON CARNIVORANS AND DIFFERENT CASE STUDIES: AGREEMENT OR DISAGREEMENT?

Taken as a whole, our results indicate that convergent evolution in the craniomandibular complex of living carnivorans is a rare phenomenon, which infrequently occurs within dietary groups. This conclusion is in good agreement with Meloro et al. (2015), who argued that, in this clade, mandibular shape is highly conserved within many dietary categories, whereas convergent evolution appears to be uncommon. Slater and Friscia (2019) also suggested that an early burst adaptive radiation characterizes the evolution of some functional ecomorphological traits (mainly related to the dentition) of extant and recently extinct terrestrial carnivorans. Early bursts imply that the evolutionary rate in a clade decreases exponentially through time, as niches are filled and ecological opportunity is exhausted (Harmon et al. 2010; Slater and Pennell 2014). Thus, pronounced divergence and rapid evolution may occur early in the adaptive radiation, whereas slower rates and conservatism would be typical of later stages. The relative rarity of convergence we found here fits well with the pioneering research of Radinsky (1981a,b), who used linear cranial measurements to explore the specializations in carnivoran hunters and concluded that they tend to involve highly idiosyncratic features. Consistently with Radinsky (1981a,b), we found the occurrence of an omnivore adaptive zone in carnivoran cranial evolution. Especially if the ancestor of this order was an omnivore, one might speculate that species with more specialized diets branched off from this generalist root and evolved in different directions and, even when they colonized similar trophic niches, only rarely converged toward similar craniomandibular shapes. In contrast, omnivores broadly retained the ancestral shape, which was already well adapted to their generalist niche.

Convergence in cases concerning either ecologically equivalent species or ecologically similar species of different body sizes (that always compared only two putatively convergent species in our analyses) was found to be a more frequent pattern as compared to convergence within dietary categories. This evidence is in line with the results obtained by Stayton (2008) analyzing a large number of datasets (simulated in the absence of functional or developmental constraints). Stayton demonstrated that, comparing multiple datasets with an equal number of species and variables, the most recurrent case of convergence is the one concerning only two observations undergoing convergent evolution across the entire tree. The simulations also pointed out that an increase in the number of species is expected to produce higher levels of convergence, whereas a greater number of variables simultaneously taken into account is likely to lower the frequency of the episodes of convergent evolution. In this sense, our study is in an intermediate position because it relies on 16 dimensions for the mandible and 28 for the cranium (i.e., numbers comparable to those employed in studies that questioned a recurrent occurrence of convergence in carnivorans or squirrels—Meloro et al. 2015; Zelditch et al. 2017), but also includes an unprecedented number of species for a research about shape convergence.

Zelditch et al. (2017) suggested that shape convergence is more likely to occur in size‐constrained niches in squirrels. The box‐whisker plots representing the size variation among our adopted dietary categories (Fig. S1) suggest a different scenario for living carnivorans: none of the ecological groups exhibits an extremely reduced size disparity and convergence could possibly have occurred only in a group (i.e., large prey hunters) that possesses an average size disparity. Nevertheless, convergence is rare in the craniomandibular shape evolution of carnivorans (as well as of squirrels), if compared with case studies such as desert lizards (Melville et al. 2006), Australian and North American snakes (Grundler and Rabosky 2014), or mainland *Anolis* (Moreno‐Arias and Calderón‐Espinosa 2016). Zelditch et al. (2017) suggested that this outcome might be produced by a complex interplay of one‐to‐many, many‐to‐one, and many‐to‐many relationships taking place between ecology, biomechanics, and morphology.

### CONCLUSIONS AND FUTURE DIRECTIONS

Convergence of craniomandibular shape rarely accompanies convergence in diet; taken together convergence and conservatism seem to have limited the disparity of Carnivora. Stayton (2008) showed that increasing taxonomic coverage is crucial for a powerful investigation of evolutionary convergence in morphology. The inclusion of a large number of small‐ and medium‐sized carnivoran species that are generally poorly sampled in ecomorphological research (e.g., viverrids that are often excluded in this field of study—Gaubert and Veron 2003; Gaubert et al. 2005) might indeed be one of the reasons that allowed us to rule out the occurrence of convergence in many dietary categories and also to validate the presence of conservatism in the omnivore group.

Nonetheless, the general lack of conclusive evidence for convergence in broad dietary groups of carnivorans does not exclude the possibility that specific cases of ecologically equivalent species might have partially converged toward similar morphologies, as confirmed by our results concerning the mandible. Results of studies of ecomorphological convergence are, however, influenced by a variety of factors, which suggest caution in interpreting them. Certainly, before trying any hypotheses on the processes behind the patterns, there is an increasingly important need to carefully test the robustness and generalizability of descriptive studies, like our and the vast majority of ecomorphological analyses.

We used multiple pattern‐based metrics designed for detecting the occurrence of retained and/or evolved similarity, each with a slightly different biological meaning. This allowed us not only to test for the presence of convergence, but also to distinguish between episodes of convergent evolution and conservatism, that are the most common processes leading to trait similarity (Moen et al. 2013). Our results support the existence of a complex relationship taking place between ecology, biomechanics, and morphology that makes convergent evolution a rare phenomenon. Ecological equivalence was the only condition that produced convergence in more than one occasion for carnivorans. Further studies about the interaction between ecological equivalence and convergence could be extremely interesting for further clarifying the mechanisms leading to a condition of evolved trait similarity. Increasing the number of studies that disprove the occurrence of convergent evolution in a specific clade, like ours, is a pivotal need in evolutionary biology and might ease the quest for previously unknown episodes of convergence.

## AUTHOR CONTRIBUTIONS

DT, CM, PR, and LM conceptualized the study. CM collected the data. DT, CM, and LM analyzed and interpreted the data. DT provided the computed code. All the authors wrote the manuscript and gave final approval of the version to be published.

## CONFLICT OF INTEREST

The authors declare no conflict of interest.

## DATA ARCHIVING

Morphological datasets, phylogeny, and R script supporting the results of this article are archived in Dryad (https://doi.org/10.5061/dryad.bg79cnpb1) and/or provided as Supporting Information.

## SSE MEMBERSHIP

DT is a member of the Society for the Study of Evolution.

Associate Editor: M. Zelditch

Handling Editor: T. Chapman.

## Supporting information

**Table S1**. Species and sample sizes.

**Figure S1**. Box‐whisker plots of natural logarithm of centroid size (lnCS) across the adopted ecological categorizations are based on differences in the main food item. Limits on boxes (light cranium, dark mandible) correspond to the first and third quartiles, whereas the internal black line represents the median.

Supporting Material
