# Supplementary material for: Testing the occurrence of convergence in the craniomandibular shape evolution of living carnivorans
Source: Evolution. 2021 May 7;75(7):1738–52. doi: 10.1111/evo.14229 (PMC8359831; doi:10.1111/evo.14229)
Supplement: Supplementary file 1 — Table S1. Species and sample sizes. [file EVO-75-1738-s001.pdf]

Table S1. Species and sample sizes.

Species	Mandible			Cranium		
	Male	Female	Unknown	Male	Female	Unknown
Acinonyx jubatus	1	1	2	1	1	2
Ailuropoda melanoleuca	0	0	1	0	0	1
Ailurus fulgens	1	2	1	1	2	1
Aonyx capensis	0	0	2	0	0	2
Aonyx cinerea	1	0	0	1	0	0
Arctictis binturong	0	2	1	0	2	1
Arctocephalus australis	1	1	3	1	1	2
Arctocephalus forsteri	2	1	0	2	1	0
Arctocephalus gazella	4	1	0	3	1	0
Arctocephalus philippii	0	0	1	0	0	1
Arctocephalus pusillus	4	5	0	4	5	0
Arctocephalus tropicalis	2	0	0	2	0	0
Arctogalidia trivirgata	1	1	0	1	1	0
Arctonyx collaris	0	0	1	0	0	1
Atelocynus microtis	3	2	0	3	1	0
Atilax paludinosus	0	1	4	0	1	4
Bassaricyon alleni	0	0	1	0	0	1
Bassariscus astutus	0	1	0	0	1	0
Bassariscus sumichrasti	1	0	0	1	0	0
Bdeogale crassicauda	0	0	2	0	0	2
Bdeogale nigripes	0	0	1	0	0	1
Callorhinus ursinus	2	3	0	2	3	0
Canis adustus	2	0	1	2	0	1
Canis aureus	2	0	1	2	0	1
Canis latrans	1	1	1	1	1	1
Canis lupus	6	2	1	6	2	1
Canis mesomelas	0	0	3	0	0	3
Canis simensis	0	0	3	0	0	6
Caracal aurata	0	1	1	0	1	1
Caracal caracal	0	0	2	0	0	2
Catopuma badia	0	1	0	0	1	0
Catopuma temminckii	0	0	1	0	0	1
Cerdocyon thous	0	0	1	0	0	1
Chrysocyon brachyurus	1	1	2	1	1	2
Civettictis civetta	2	6	2	2	4	2
Conepatus chinga	1	0	0	1	0	0
Conepatus leuconotus	0	0	1	0	0	1
Crocuta crocuta	1	0	6	0	0	6
Cryptoprocta ferox	2	0	0	2	0	0
Cuon alpinus	0	0	2	0	0	2
Cynictis penicillata	0	0	2	0	0	1
Cystophora cristata	0	1	1	0	1	1
Dusicyon australis	1	0	0	0	0	3
Eira barbara	1	0	2	1	0	2
Enhydra lutris	0	0	2	0	0	3
Erignathus barbatus	0	0	1	0	0	1
Eumetopias jubatus	2	1	1	1	1	1

Species	Mandible			Cranium		
	Male	Female	Unknown	Male	Female	Unknown
Felis chaus	1	0	0	1	0	1
Felis margarita	1	1	0	1	1	0
Felis nigripes	1	1	0	1	1	0
Felis silvestris	4	6	2	3	6	1
Galictis cuja	0	0	1	0	0	1
Galictis vittata	1	0	2	1	0	1
Galidia elegans	0	0	2	0	0	2
Galidictis fasciata	0	0	4	0	0	4
Genetta angolensis	3	0	0	3	0	0
Genetta genetta	0	0	1	0	0	1
Genetta maculata	3	2	1	3	2	1
Genetta servalina	2	0	0	2	0	0
Genetta tigrina	1	0	1	1	0	1
Genetta victoriae	0	0	1	0	0	1
Gulo gulo	0	0	4	0	0	4
Halichoerus grypus	2	3	0	3	3	0
Helarctos malayanus	1	1	2	0	1	2
Helogale parvula	0	0	3	0	0	3
Hemigalus derbyanus	0	1	0	0	1	0
Herpailurus yagouaroundi	0	0	1	0	0	1
Herpestes auropunctatus	0	0	1	0	0	1
Herpestes edwardsii	0	0	1	0	0	1
Herpestes ichneumon	0	1	2	0	1	2
Herpestes javanicus	0	0	1	0	0	1
Herpestes naso	0	0	1	0	0	1
Herpestes sanguineus	0	1	3	0	1	3
Herpestes smithii	0	0	1	0	0	1
Herpestes vitticollis	0	0	1	0	0	1
Histriophoca fasciata	2	1	0	2	1	0
Hyaena hyaena	0	2	3	0	2	3
Hydrictis maculicollis	0	0	1	0	0	1
Ichneumia albicauda	0	1	2	0	1	1
Ictonyx striatus	1	1	1	1	1	0
Leopardus colocolo	0	0	1	0	0	1
Leopardus geoffroyi	0	1	1	0	1	1
Leopardus guigna	0	0	1	0	0	1
Leopardus pardalis	1	0	1	1	0	2
Leopardus wiedii	0	1	0	0	0	1
Leptailurus serval	2	1	0	2	1	1
Leptonychotes weddellii	1	2	0	2	2	0
Lobodon carcinophaga	2	2	0	2	2	0
Lontra canadensis	1	0	0	1	0	0
Lontra felina	0	0	1	0	0	1
Lontra longicaudis	0	0	1	0	0	1
Lontra provocax	0	0	1	0	0	1
Lutra lutra	1	0	1	1	0	1
Lycalopex culpaeus	0	0	3	0	0	2

Species	Mandible			Cranium		
	Male	Female	Unknown	Male	Female	Unknown
Lycalopex fulvipes	0	0	1	0	0	1
Lycalopex griseus	1	0	7	1	0	5
Lycalopex gymnocercus	1	1	0	1	1	0
Lycalopex vetulus	1	1	0	1	1	0
Lycaon pictus	1	2	4	1	2	4
Lynx canadensis	2	2	1	2	1	1
Lynx lynx	0	1	1	0	1	1
Lynx pardinus	0	0	1	0	0	1
Lynx rufus	0	0	1	0	0	1
Martes americana	0	0	1	0	0	1
Martes flavigula	0	0	1	0	0	1
Martes foina	2	0	0	2	0	0
Martes martes	0	1	2	0	1	2
Martes melampus	1	0	0	1	0	0
Martes pennanti	1	1	0	1	1	0
Meles meles	8	5	0	4	4	0
Mellivora capensis	1	0	2	1	0	1
Melogale moschata	1	1	1	1	1	1
Melursus ursinus	2	2	1	2	2	1
Mephitis macroura	1	0	0	0	1	0
Mephitis mephitis	0	1	0	0	1	0
Mirounga leonina	2	1	0	2	1	0
Monachus monachus	2	1	0	2	1	0
Mungos mungo	2	2	1	2	1	1
Mustela erminea	2	0	3	2	0	2
Mustela frenata	0	0	1	0	0	1
Mustela lutreola	0	0	1	0	0	1
Mustela nivalis	2	1	1	1	1	1
Mustela putorius	2	1	2	2	1	2
Mustela sibirica	0	0	1	0	0	1
Nandinia binotata	3	3	0	3	3	0
Nasua narica	0	0	2	0	0	2
Nasua nasua	0	0	5	0	0	3
Neofelis nebulosa	1	1	0	1	1	0
Neophoca cinerea	2	1	1	2	1	1
Neovison vison	5	1	1	5	1	1
Nyctereutes procyonoides	1	1	1	1	1	1
Odobenus rosmarus	1	0	1	0	0	3
Ommatophoca rossii	1	0	1	1	0	1
Otaria byronia	3	1	0	3	1	0
Otocyon megalotis	3	1	2	2	1	2
Pagophilus groenlandicus	2	1	2	2	1	1
Paguma larvata	0	0	1	0	0	1
Panthera leo	5	3	2	5	3	1
Panthera onca	2	1	1	2	1	1
Panthera pardus	0	2	6	0	2	5
Panthera tigris	1	2	1	1	2	1

Species	Mandible			Cranium		
	Male	Female	Unknown	Male	Female	Unknown
Panthera uncia	0	1	1	0	1	1
Paradoxurus hermaphroditus	0	0	1	0	0	1
Parahyaena brunnea	0	0	2	0	0	2
Pardofelis marmorata	0	1	0	0	1	0
Phoca largha	1	1	0	1	1	0
Phoca vitulina	0	0	3	0	0	3
Phocarctos hookeri	3	0	0	3	0	0
Poecilogale albinucha	0	0	2	0	0	2
Potos flavus	1	1	0	1	1	0
Prionailurus bengalensis	0	0	3	0	0	3
Prionailurus planiceps	0	1	0	0	1	0
Prionailurus rubiginosus	0	0	1	0	0	1
Prionailurus viverrinus	0	0	2	0	0	2
Prionodon linsang	0	0	1	0	0	1
Procyon cancrivorus	0	1	1	0	1	2
Procyon lotor	1	1	1	1	1	1
Proteles cristata	1	0	2	1	0	2
Pteronura brasiliensis	2	0	0	2	0	0
Puma concolor	0	1	1	0	1	1
Pusa caspica	0	2	0	0	2	0
Pusa hispida	2	1	1	2	1	1
Pusa sibirica	1	1	0	1	1	0
Rhynchogale melleri	0	0	1	0	0	1
Salanoia concolor	1	0	2	1	0	1
Speothos venaticus	1	1	0	1	1	0
Spilogale putorius	0	0	1	0	0	1
Suricata suricatta	0	0	1	0	0	1
Taxidea taxus	0	0	1	0	0	2
Tremarctos ornatus	3	0	0	2	0	0
Urocyon cinereoargenteus	2	2	2	2	1	2
Urocyon littoralis	0	0	1	0	0	1
Ursus americanus	2	2	2	2	2	2
Ursus arctos	3	4	4	3	4	4
Ursus maritimus	0	0	5	0	0	4
Ursus thibetanus	1	1	1	1	1	1
Viverra tangalunga	2	0	0	2	0	0
Viverra zibetha	0	1	0	0	0	1
Viverricula indica	0	0	5	0	0	5
Vulpes cana	1	0	0	1	0	0
Vulpes chama	1	2	1	1	2	1
Vulpes corsac	0	0	2	0	0	1
Vulpes ferrilata	2	0	1	2	0	1
Vulpes lagopus	2	2	6	2	2	6
Vulpes rueppellii	0	1	0	0	1	0
Vulpes vulpes	6	5	0	6	5	0
Vulpes zerda	1	0	1	1	0	0
Zalophus californianus	2	1	0	2	1	0
