# Supplementary material for: Testing the occurrence of convergence in the craniomandibular shape evolution of living carnivorans
Source: Evolution. 2021 May 7;75(7):1738–52. doi: 10.1111/evo.14229 (PMC8359831; doi:10.1111/evo.14229)
Supplement: Supplementary file 2 — Figure S1. Box‐whisker plots of natural logarithm of centroid size (lnCS) across the adopted ecological categorizations are based on differences in the main food item. Limits on boxes (light cranium, dark mandible) correspond to the first and third quartiles, whereas the internal black line represents the median. [file EVO-75-1738-s002.pdf]

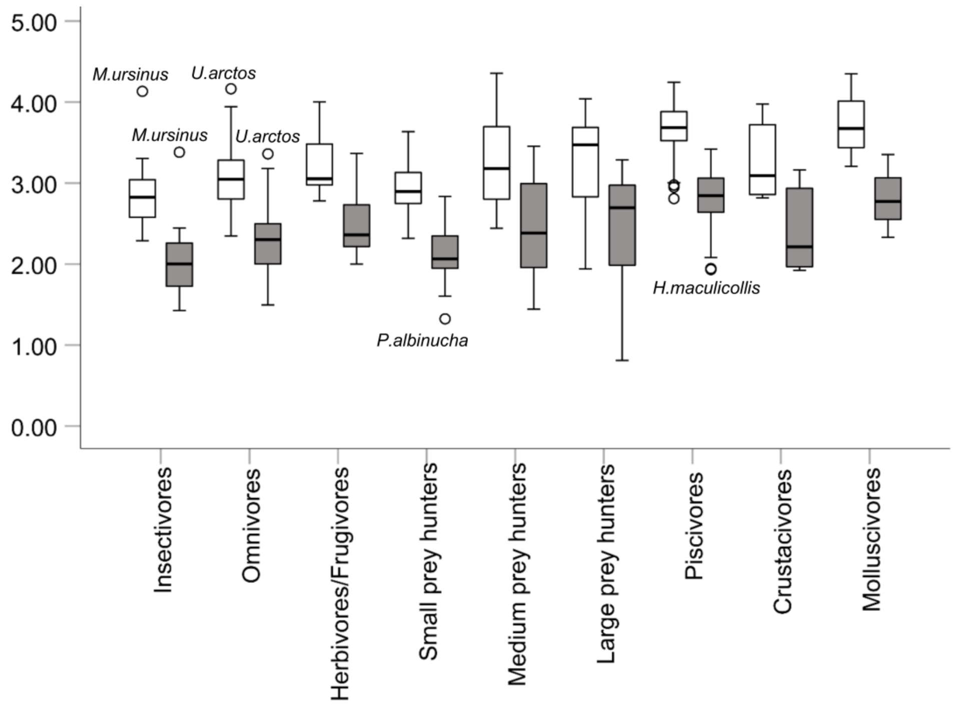

Figure S1 Box-whisker plots of natural logarithm of centroid size (lnCS) across the adopted ecological categorisations are based on differences in the main food item. Limits on boxes (light cranium, dark mandible) correspond to the first and third quartiles, while the internal black line represents the median. Whiskers extend from minimum to maximum values. Circles represent outliers.
